# Closing the Mad2 cycle

**DOI:** 10.7554/eLife.08283

**Published:** 2015-05-22

**Authors:** Andrea Musacchio

**Affiliations:** Department of Mechanistic Cell Biology, Max Planck Institute of Molecular Physiology, Dortmund, Germany and the Centre for Medical Biotechnology, Faculty of Biology, University Duisburg-Essen, Essen, Germanyandrea.musacchio@mpi-dortmund.mpg.de

**Keywords:** checkpoint proteins, spindle assembly checkpoint, AAA+ ATPase, HORMA domain protein, Mad2, *C. elegans*, mouse

## Abstract

Chromosome separation is regulated by a cycle that involves a protein undergoing an unusual topological conversion.

**Related research article** Ye Q, Rosenberg SC, Moeller A, Speir JA, Su TY, Corbett KD. 2015. TRIP13 is a protein-remodeling AAA+ ATPase that catalyzes MAD2 conformation switching. *eLife* **4**:e07367. doi: 10.7554/eLife.07367

**Image** The three-dimensional structure of the enzyme TRIP13/PCH-2

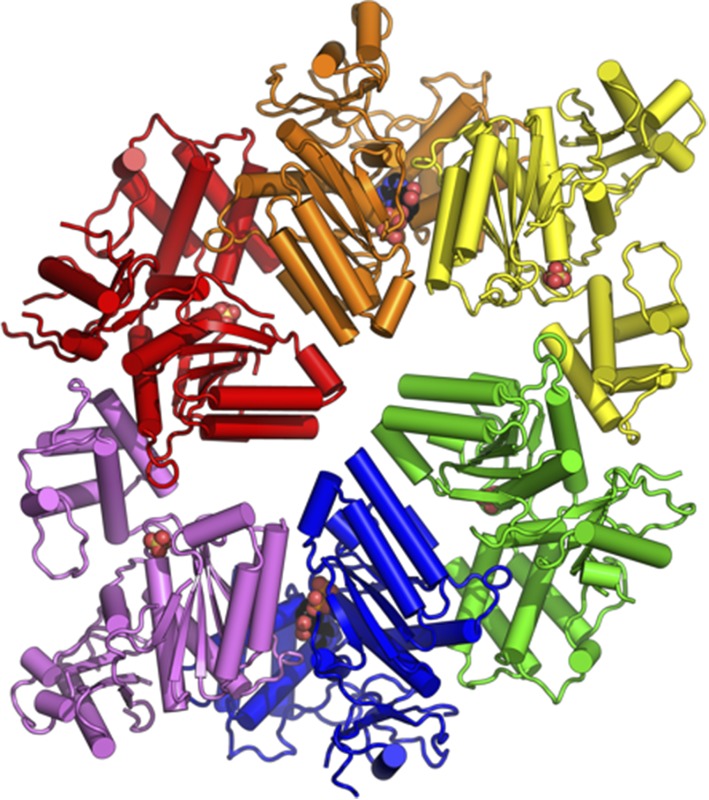

Enzymes and other proteins constantly undergo changes of conformation in our cells. In most cases these changes do not involve any change in the topology of the protein: that is, the relative positions of the secondary structure elements of the protein (such as alpha helices and beta sheets) do not change. A family of proteins called HORMA domain proteins has emerged as a fascinating exception to this empirical rule. Mad2, the best-studied member of this family, changes between an open state that is inactive and a closed (active) state with a different topology (Figure 1). Crucial details of this process have remained unclear but now, in *eLife*, Kevin Corbett and co-workers—including Qiaozhen Ye as first author—shed new light on the role played by an enzyme called TRIP13/PCH-2 in this particular conformational change (Ye et al., 2015).

**Figure 1.**
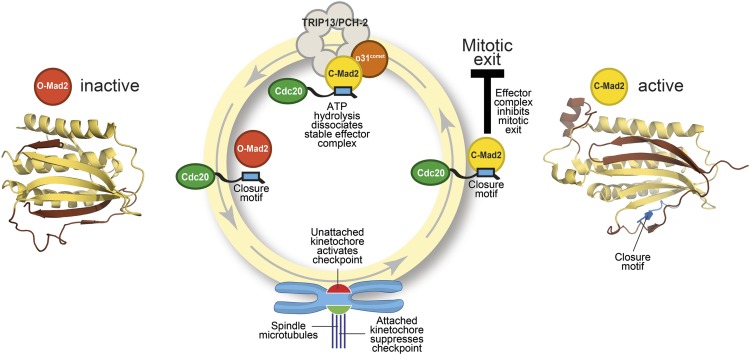
The Mad2 cycle. The Mad2 protein exists in an ‘inactive’ open form (left) and an ‘active’ closed form (right). The structural elements highlighted in pale yellow have the same relative positions in the two states; the elements highlighted in brown have different relative positions. The open form (red) is converted to the closed form (bright yellow) by a closure motif (blue rectangle) within a Cdc20 protein. This process is strongly promoted by kinetochores that have not yet bound to the spindle (red), but not by those that are already bound (green). This results in the formation of the effector complex (containing closed Mad2 and Cdc20) that suppresses the separation of the chromosomes. A protein called p31^comet^ acts as a bridge to allow an enzyme called TRIP13/PCH-2 to use ATP hydrolysis to dissociate this effector complex, which is very stable.

Before a cell divides, its chromosomes need to be duplicated and then separated into two groups so that each daughter cell gets a full set of chromosomes. To achieve this, protein complexes called kinetochores connect the chromosomes to a structure called the spindle, which pulls the chromosomes to opposite ends of the cell. This process is monitored by a set of proteins known as checkpoint proteins.

Mad2 is a checkpoint protein that is recruited in its open (inactive) form to kinetochores that are not yet properly attached to the spindle. It is converted to the closed (active) form by binding to a ‘closure motif’ in a protein called Cdc20. The closed Mad2 protein then joins forces with Cdc20 and other proteins to create a checkpoint effector complex that prevents the chromosomes separating until all of them are attached to the spindle (Figure 1).

When purified samples of Mad2 and Cdc20 are mixed in the laboratory, they bind together spontaneously. However, this process is very slow because a large amount of activation energy is needed to convert the open state of Mad2 into the closed state. Inside the cell, however, the kinetochores act as catalysts to accelerate the reaction through steps that are only partly understood (Lara-Gonzalez et al., 2012).

Several lines of evidence indicate that closed Mad2 can be converted back into the open form. Moreover, even when the checkpoint effector is active, some open Mad2 is always present: this allows the open form of the protein to be recruited to kinetochores that are not yet attached to the spindle. This pool of open Mad2 is not maintained through the production of new protein, so the most plausible explanation is that it comes from the continuous conversion of closed Mad2. How does this take place? Previous studies have established that the hydrolysis of ATP is required to disassemble the checkpoint effector (Miniowitz-Shemtov et al., 2010): hydrolysis of ATP releases energy, but the specific steps that require this energy had not been identified.

The key to gaining a molecular understanding was the characterization of two proteins—TRIP13/PCH2 and p31^comet^—that were known to be involved in disassembling the effector complex formed by closed Mad2 and Cdc20 (Eytan et al., 2014; Wang et al., 2014). By recreating the reaction in the laboratory with purified components, Ye et al.—who are based at the Ludwig Institute for Cancer Research, the Scripps Institute and the University of California, San Diego—now go further by showing that the TRIP13/PCH-2 enzyme binds to closed Mad2, which is then converted to open Mad2 in a manner that depends on ATP hydrolysis and on the activity of p31^comet^ (Figure 1).

TRIP13/PCH-2 is a member of the AAA+ ATPase family of enzymes: these enzymes, which are found in all kingdoms of life, harness the chemical energy released by ATP hydrolysis to perform mechanical work in a variety of different reactions (Hanson and Whiteheart, 2005; Vader, 2015). For instance, an important subclass of these enzymes—known as the disaggregases—can separate proteins that have formed aggregates within the cell: this allows these proteins to refold with assistance from chaperone proteins. Another subclass assists in the breakdown of proteins by associating with protease enzymes, and a third subclass promotes the disassembly of stable protein complexes and the recycling of their subunits. TRIP13/PCH-2 appears to belong to the latter class, but Ye et al. reveal that its high-resolution three-dimensional structure is more typical of the disaggregases.

As if the pattern of events surrounding the conversion of Mad2 had not been complex enough, p31^comet^ is also a member of the HORMA domain family and is able to selectively interact with closed Mad2 (Yang et al., 2007). Ye et al. now demonstrate that p31^comet^ binds to Mad2 and TRIP13/PCH2 at the same time, and acts as a bridge between them (Figure 1).

TRIP13/PCH-2 can also regulate a group of HORMA domain proteins that are involved at multiple stages of a type of cell division called meiosis (Vader, 2015). The significance of this regulation was not clear at first, but recent studies have revealed that two of these HORMA domain proteins interact with the closure motifs in their binding partners, indicating that their safety belt is, like that of Mad2, a variable element of their topology (Chen et al., 2014; Kim et al., 2014).

Collectively, these recent studies show that TRIP13/PCH-2 is a general regulator of a topological conversion that occurs when HORMA domains bind to their partner proteins. The techniques described by Corbett, Ye and co-workers are without doubt a promising way forward for the further investigation of this fascinating problem.
